# METTL3-mediated methylation of CYP2C19 mRNA may aggravate clopidogrel resistance in ischemic stroke patients

**DOI:** 10.1515/med-2024-0899

**Published:** 2024-02-07

**Authors:** Quandan Tan, Le Yang, Shanshan Yuan, Danni Zheng, Yapeng Lin, Kejie Chen, Ying He, Shuntian Chen, Junli Hao, Jin Dai, Song He, Fengkai Mao, Xinyi Leng, Haisong Jiang, Jie Yang

**Affiliations:** Department of Neurology, The First Affiliated Hospital of Chengdu Medical College, Chengdu, China; Department of Neurology, The Second Affiliated Hospital of Chengdu Medical College, Chengdu, China; Department of Critical Care Medicine, The General Hospital of Western Theater Command, Chengdu, China; Biomedical Informatics and Digital Health, School of Medical Sciences, University of Sydney, Sydney, Australia; International Clinical Research Center, Chengdu Medical College, Chengdu, China; School of Public Health, Chengdu Medical College, Chengdu, China; Department of Rheumatology and Immunology, The First Affiliated Hospital of Chengdu Medical College, Chengdu, China; School of Biomedical Sciences and Technology, Chengdu Medical College, Chengdu, China; Department of Medicine & Therapeutics, The Chinese University of Hong Kong, Hong Kong, China; Department of Neurology, Sichuan Provincial People’s Hospital, School of Medicine, University of Electronic Science and Technology of China, Chengdu, China

**Keywords:** N6-methyladenosine, METTL3, CYP2C19, clopidogrel resistance, ischemic stroke

## Abstract

**Background:**

N6-methyladenosine (m^6^A) is the most frequently occurring interior modification in eukaryotic messenger RNA (mRNA), and abnormal mRNA modifications can affect many biological processes. However, m^6^A’s effect on the metabolism of antiplatelet drugs for the prevention of ischemic stroke (IS) remains largely unclear.

**Methods:**

We analyzed the m^6^A enzymes and m^6^A methylation in peripheral blood samples of IS patients with/without clopidogrel resistance (CR), and the peripheral blood and liver of rat models with/without CR. We also compared the effect of m^6^A methylation on the expression of the drug-metabolizing enzymes (CYP2C19 and CYP2C6v1) in CR and non-CR samples.

**Results:**

Methyltransferase-like 3 (METTL3), an m^6^A enzyme, was highly expressed in the peripheral blood of patients with CR, and in both the peripheral blood and liver of rats with CR. This enzyme targets CYP2C19 or CYP2C6v1 mRNA through m^6^A methylation, resulting in low expression of CYP2C19 or CYP2C6v1 mRNA. Consequently, this leads to decreased clopidogrel metabolism and CR.

**Conclusion:**

The METTL3-mediated methylation of CYP2C19 mRNA may aggravate CR in IS patients.

## 1 Introduction

The global burden of stroke is large and escalating, making it a leading cause of adult disability and death worldwide [1,2]. Alongside this, the incidence of recurrent stroke continues to rise [3]. Antiplatelet therapy has been shown to reduce annual stroke risk by 9% in patients with carotid artery disease and prevents stroke recurrence in 20% of patients with ischemic stroke (IS) [4,5]. Clopidogrel is one of the most studied and widely used antiplatelets for the prevention of IS, and trials have shown that clopidogrel can effectively reduce serious cerebrovascular events, such as stroke recurrence [6,7]. However, 17.2–86.1% of patients receiving clopidogrel have shown inadequate treatment response and experienced recurrent IS or other vascular events in the Asian population [8], which is termed clopidogrel resistance (CR) [9]. The mechanisms of CR remain largely unclear [10]. Hepatic CYP2C19 is the key hepatic enzyme that converts clopidogrel to its active form [8,11]. Research into new CYP2C19 and CR regulating mechanisms is urgently needed.

N6-methyladenine (m^6^A) modification is an epitranscriptomic modification, considered to be the most prevailing and reversible modification of messenger RNA (mRNA) in eukaryotes [12]. Methyltransferase-like 3 (METTL3) is an mRNA methyltransferase that has been shown to be a writer of m^6^A [13], and METTL3 can participate in mRNA biogenesis, translation, and decay through m^6^A methylation [14]. Previous studies [15–18] have demonstrated that METTL3 plays an important role in anticancer drug resistance, particularly in regulating the expression of cytochrome P450 family members [18]. Nevertheless, the impact of METTL3 on antiplatelet drug resistance is unclear. An in-depth and comprehensive elucidation of the m^6^A mechanisms of CYP2C19 regulation and CR may help guide individualized clopidogrel therapy for IS patients.

## 2 Materials and methods

### 2.1 Human samples

Human sample studies were conducted according to the principles of the Declaration of Helsinki and authorized by the Ethics Supervision Committee of Sichuan Provincial People’s Hospital (Ethics No. 251, 2022). The inclusion criteria were (1) participants aged 18–75 years, (2) patients diagnosed with IS, and (3) those who had been taking clopidogrel 75 mg/day continuously as the sole antiplatelet agent for more than 5 days. The exclusion criteria were (1) patients receiving anticoagulation and thrombolytic therapy, (2) patients taking other antiplatelet drugs, (3) patients with malignant tumors and liver diseases, and (4) patients with severe organ failure. Written informed consent was attained from the patients or legal guardians of subjects participating in this study. Platelet aggregation function tests were performed in subjects using VerifyNow (Accumetrics Inc., San Diego, USA). CR was defined by a P2Y12 Reaction Units (PRU) test value greater than 208 [19–21].

### 2.2 Animal experiments

All animal experiments are authorized by the Laboratory Animal Welfare Ethics Review Committee of Chengdu Medical College (IACUC-22-026). Sprague-Dawley rats (SPF grade, >400 g, male, 3 months old), purchased from Chengdu Dashuo Laboratory Animal Co., Ltd, were adapted to standard housing conditions for 1 week under a 12 h light/12 h dark cycle.

Clopidogrel (1 mg/kg/day, Shenzhen Xinlitai Pharmaceutical Co., Ltd, China) powder was dissolved in saline and administered intragastrically to rats for 4 days. On the night of the fourth day, rats were food-deprived but allowed to drink. On the morning of the fifth day, rats were anesthetized with sodium pentobarbital (50 mg/kg body weight, i.p.), followed by jugular venous blood collection. About 1.8 mL of blood was collected into a single-use vacuum blood collection containing sodium citrate (Huabo Medical Device Co., Ltd, China) and the test tube was reversed five times to intermix the contents. Automated platelet aggregation (PL-12, SINNOWA, China) was used to detect adenosine diphosphate (ADP) (Sigma-Aldrich, Deisenhofen, Germany)-mediated platelet aggregation, including platelet count, average aggregation rate, and maximum aggregation rate (MAR). CR was defined as having a platelet aggregation inhibition rate ([unadministered MAR – MAR after administration]/unadministered MAR) <30% [22].

The rats were fully perfused with 1× phosphate buffered saline (PBS) solution. Following this, the hepatic portal duct system was ligated and severed using blunt and sharp combination method. The liver tissue was placed in sterile Petri dishes, washed with 1× PBS solution, and then weighed and recorded. The rat liver tissue was divided into five parts on average, one part was used for the experiment, and the rest was put into a sterile frozen storage tube at −80°C for storage.

### 2.3 Western blot (WB)

Lysis buffer (Real-Times (Beijing) Biotechnology Co., Ltd, RL1020) was used to lyse peripheral blood and liver tissue into protein, and the supernatant fluid was collected for western blotting. Regarding homogenization methods, we used a glass homogenizer to grind up the liver tissue of rats. Before analysis, the protein concentration was measured using a BCA kit (Beyotime Biotechnology, China). First, the same quantity of protein was subjected to sodium dodecyl sulfate-polyacrylamide gel electrophoresis and transferred to polyvinylidene fluoride membranes. Membranes were blocked with 5% defatted milk for 1 h on a shaking table and incubated by primary anti-METTL3 antibody (Proteintech, #27226-1-AP, 1:1,000), anti-CYP2C19 antibody (Monoclonal, 10G5, 1:1,500), and anti-GAPDH (Proteintech, #60004-1-Ig, 1:10,000) for 75 min at room temperature. We washed the membranes with PBST buffer three times, 5 min each time, and incubated the membranes with an appropriate secondary antibody for 1 h at room temperature. After that, membranes were scanned and imaged promptly. All experimentations were performed three times, and typical blots were displayed.

### 2.4 Quantitative RT-PCR

Total RNA was extracted from peripheral blood and liver tissue with TRIzol reagent (Invitrogen, USA). After that, the RNA was reverse-transcribed to cDNA according to the manufacturer’s (EZBioscience, USA) instructions. The relative expression of objective genes was standardized by GAPDH and computed by the 2^−ΔΔT^ method. The primer sequences utilized in this study are listed in Table 1.

**Table 1:** Primer sequence

Gene primers	Sequences (5′–3′)
Human-METTL3-F	GTTAGCCTTCGGGGTGTCC
Human-METTL3-R	GTAGATCCAAGTGCCCCGAG
Human-METTL14-F	TTGGACCTTGGAAGAGTGTGTTT
Human-METTL14-R	TGAAGTCCCCGTCTGTGCTA
Human-FTO-F	AATTCTATCAGCAGTGGCAGC
Human-FTO-R	TGAGGATGCGAGATACCGGA
Human-ALKBH5-F	GTGCTCAGTGGATATGCTGC
Human-ALKBH5-F	TTGGGTTTCAGAGCAGGGTC
Human-CYP2C19-F	AGGATTGTAAGCACCCCCTG
Human-CYP2C19-R	TGTCCATCGATTCTTGGTGTTC
Human-GAPDH-F	GAAAGCCTGCCGGTGACTAA
Human-GAPDH-R	GCCCAATACGACCAAATCAGAGA
Rat-METTL3-F	ATGTGCAGCCCAACTGGATT
Rat-METTL3-R	CTGTGCTTAAACCGGGCAAC
Rat-CYP2C6v1-F	TTTGAGCAGTCCCTGGACAC
Rat-CYP2C6v1-R	AGTCCCGGGGATTTGTAACAT
Rat-GAPDH-F	TCTCTGCTCCTCCCTGTTCT
Rat-GAPDH-R	TACGGCCAAATCCGTTCACA

### 2.5 Dot blot

RNA (1 µg) was spotted onto a nitrocellulose membrane (PR04769, Merck Millipore Ltd, Tullagreen, Carrigtwohill, Co. Cork, Ireland). Subsequently, the membrane was UV-crosslinked and blocked in tris buffered saline with Tween-20 (TBST), 0.1% Tween 20 plus TBS (50 mM Tris-Cl, 150 mM NaCl, pH 7.5), which contained 5% bovine serum albumin at room temperature for 1 h. M^6^A monoclonal antibody was diluted at 1:2,000 with TBS-T, incubated at 4°C overnight, and then washed three times with TBST. Rabbit anti-mouse IgG H&L (HRP) was prepared by diluting it at a ratio of 1:10,000, and solution was incubated for 1 h at room temperature. Following incubation, it was washed three times with TBST and incubated with enhanced chemiluminescence reagents for 1 min. After incubation, we covered it with plastic wrap, ensuring to remove any excess solution from the surface. Finally, the X-ray film was exposed in the darkroom.

### 2.6 Prediction of m^6^A sites

We used SRAMP (sequence-based RNA adenosine methylation site predictor) (http://www.cuilab.cn/sramp.) to predict the m^6^A methylation site of METTL3 mRNA. Only RNA sequences are required when running a prediction and no external omics data were loaded. We selected “Mature mRNA mode,” then completed all steps according to the prompts, and finally clicked “Submit” to get the predicted results.

### 2.7 Methylated RNA immunoprecipitation (MeRIP)

After total RNA (200 µg) was extracted with TRIzol reagent, RiboMinusTM Eukaryote Kit v2 (A15020, Invitrogen) was used to remove ribosomal RNA. Afterward, the RNA was cut into about 100 nucleotide fragments using RNA fragmentation reagents (AM8740, Invitrogen). Subsequently, a part of the RNA liquor as input for PCR or RNA sequencing was preserved at –80°C. An anti-m^6^A antibody (Abcam) was incubated with RNA at 4°C for 1 h. Prewashed PierceTM Protein A/G Magnetic Beads (88803, Thermo Scientific) were intermixed with the antibody-treated RNA in immunoprecipitation buffer overnight at 4°C. At last, the methylated RNA was purified for qPCR.

### 2.8 Statistical analysis

GraphPad Prism software (Version 9, GraphPad Software, Inc., USA) was used for data analysis. Outcomes were presented as mean ± standard deviation, and the differences between the two groups were analyzed by Student’s *t*-test. When the *P*-value was less than 0.05, the difference was regarded as statistically significant.

## 3 Results

### 3.1 METTL3 levels in the peripheral blood of CR patients are elevated

**Figure 1:**
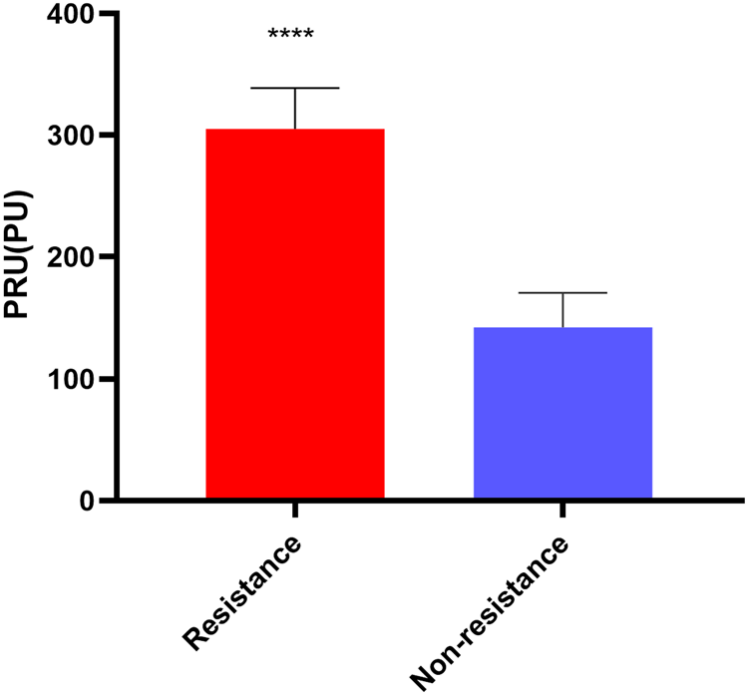
PRU of human blood samples. CR was defined as having a test result >208 P2Y12 Reaction Units (PU). Values are mean ± std. *N* = 5. Unpaired *t*-test, ****P < 0.0001.

**Figure 2:**
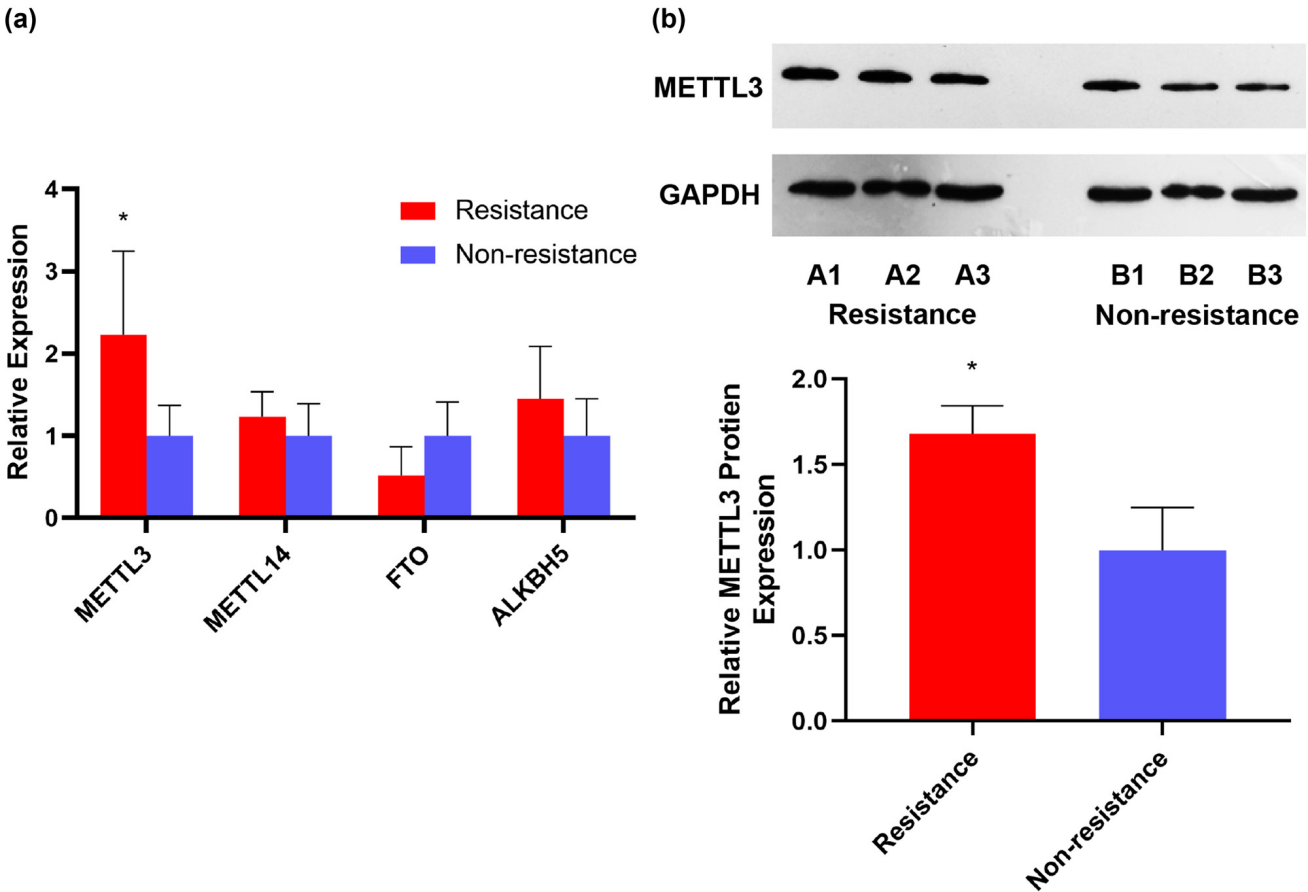
METTL3 levels in peripheral blood were elevated in humans with CR. (a) qPCR test results of m^6^A regulators expression levels in mRNA. (b) WB analysis results of METTL3 expression levels. Values are mean ± std. *N* = 3. Unpaired *t*-test, *P < 0.05.

We collected blood samples from ten IS patients (including five CR patients) (Figure 1) (specific details can be found in Section 2). In the peripheral blood samples of the human CR and non-CR groups, we measured changes in the expression levels of regulators that control RNA m^6^A modifications, including METTL3, METTL14, FTO, and ALKBH5. The results of qPCR demonstrated that the expression levels of METTL3 in the peripheral blood from the human CR group were up-regulated 2.2-fold compared to the non-CR group (Figure 2a). In addition, the results of the western blotting showed that the expression levels of METTL3 in the peripheral blood from the human CR group were up-regulated 1.7-fold compared to the non-CR group (Figure 2b). The results of western blotting were also in keeping with those of qPCR. Thus, in subsequent studies, METTL3 was selected as the primary m^6^A methylation-related molecule.

### 3.2 METTL3 modifies CYP2C19 mRNA m^6^A in the peripheral blood of CR patients, which is associated with a decrease in CYP2C19 mRNA and protein

**Figure 3:**
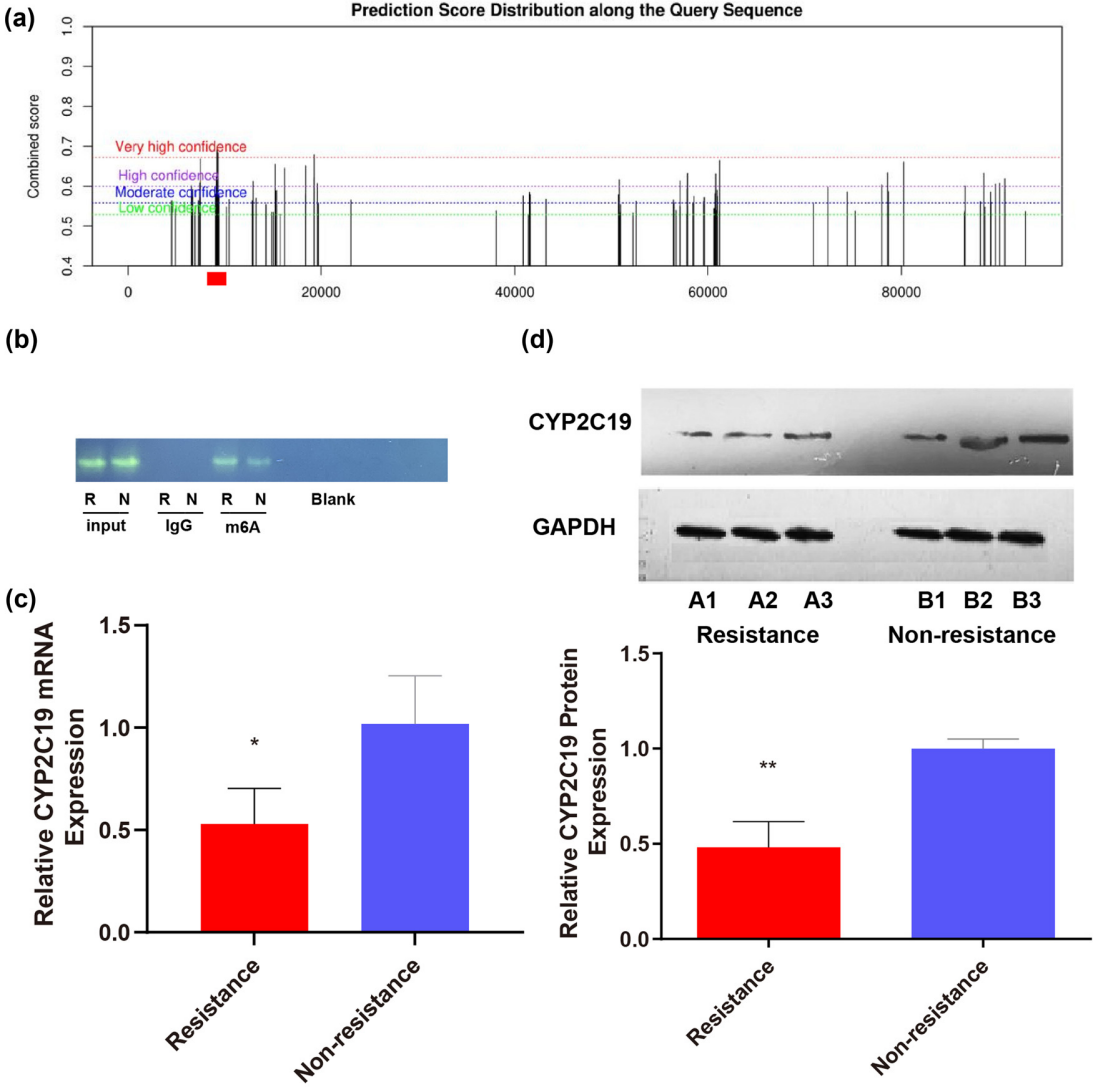
METTL3 modifies CYP2C19 RNA m^6^A in the peripheral blood of human subjects with CR, which is associated with decreased CYP2C19 RNA and CYP2C19 expression. (a) Prediction of the possible m^6^A methylation site of CYP2C19 mRNA. (b) MeRIP results. M^6^A methylation levels of CYP2C19 mRNA in the peripheral blood of human clopidogrel resistant is higher than that of non-clopidogrel resistant. (c and d) The levels of CYP2C19 in human peripheral blood were determined by qPCR and WB analysis. Values are mean ± std. *N* = 3. Unpaired *t*-test, *P < 0.05, **P < 0.01. R, resistance; N, non-resistance.

To verify whether CYP2C19 mRNA itself is affected by RNA m^6^A methylation, we predicted the possible m^6^A methylation site of CYP2C19 mRNA and performed a MeRIP test (Figure 3a). We found that the complex cross-link with the anti-m^6^A antibody of CYP2C19 in the CR group can be amplified to obtain a specific product of CYP2C19 mRNA, which is more than that in the non-CR group (Figure 3b). The experimental results showed higher levels of m^6^A methylation of CYP2C19 mRNA in the peripheral blood of CR patients compared to non-CR patients. Subsequently, the results of qPCR revealed that the expression levels of CYP2C19 mRNA in the peripheral blood of the CR group were down-regulated 0.53-fold compared to that of the non-CR group (Figure 3c). The results of WB revealed that the expression levels of CYP2C19 protein in the peripheral blood of the CR group were down-regulated 0.48-fold compared to that of the non-CR group (Figure 3d). Hence, the experimental results indicated that in patients with CR, METTL3 promoted m^6^A methylation at specific sites in CYP2C19 mRNA. This increased methylation correlates with decreased expression of CYP2C19 mRNA and CYP2C19, which in turn may lead to reduced clopidogrel metabolism.

### 3.3 Elevated METTL3 leads to increasing global m^6^A levels in rats with CR

**Figure 4:**
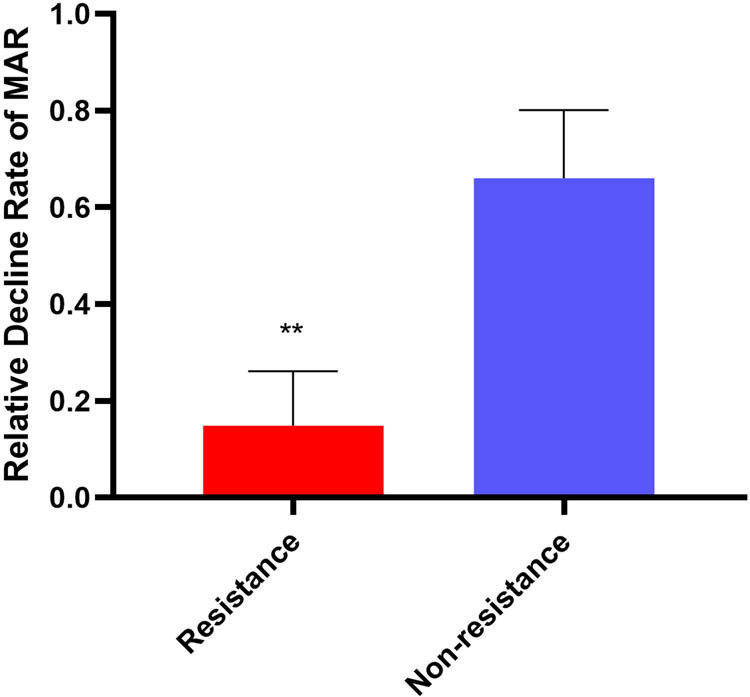
Relative decline rate of MAR in the peripheral blood of rats. CR is defined as platelet aggregation inhibition rate = (unadministered MAR – MAR after administration)/unadministered MAR <30%. Values are mean ± std. *N* = 3. Unpaired *t*-test, **P < 0.001.

**Figure 5:**
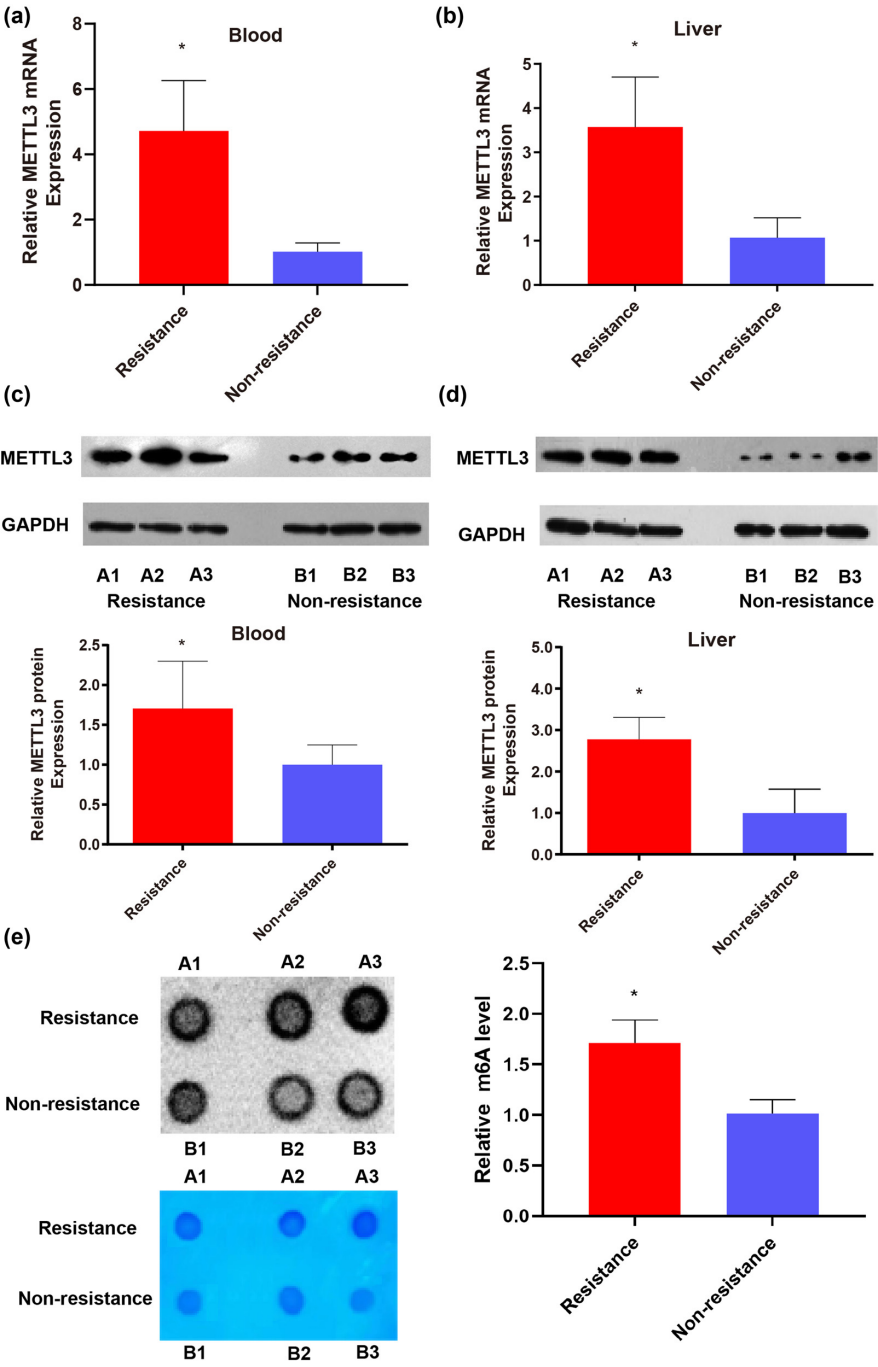
METTL3 levels in peripheral blood and liver were elevated in rats with CR. (a) qPCR results of METTL3 expression levels in mRNA in peripheral blood of rats. (b) qPCR results of METTL3 expression levels in mRNA in the liver of rats. (c) WB results of METTL3 protein expression in peripheral blood of rats. (d) WB results of METTL3 protein expression in the liver of rats. (e) Dot blot analysis results of global m^6^A levels in the liver of rats. Values are mean ± std. *N* = 3. Unpaired *t*-test, *P < 0.05.

Six rats (three CR and three non-CR) were used for experimentation (Figure 4) (specific details can be seen in Section 2). We compared the peripheral blood and liver expression levels of METTL3 between rats with CR and non-CR. The results of qPCR and WB revealed that the METTL3 levels in the peripheral blood and liver of rats in the CR group were dramatically elevated compared with rats in the non-CR group (Figure 5a–d). The results of qPCR demonstrated that the expression levels of METTL3 mRNA with the CR group were up-regulated 4.7-fold in the peripheral blood of rats and 3.6-fold in the liver of rats compared to the non-CR group (Figure 5a and b). The results of WB showed that the expression levels of METTL3 were up-regulated 1.7-fold in the peripheral blood of rats and 2.8-fold in the liver of rats compared to the control (Figure 5c and d). These results were in keeping with the results from the analysis of CR human peripheral blood. METTL3 levels were elevated both in IS patients with CR and in rats with CR. The results of the dot blot test in the liver of rats revealed that the global m^6^A levels in the CR group were up-regulated 1.7-fold compared to the non-CR group (Figure 5e). Together, these results imply that elevated levels of m^6^A methylation in the liver of rats with CR are primarily associated with elevated METTL3.

### 3.4 METTL3 modifies CYP2C6v1 mRNA in the peripheral blood and liver of rats with CR and results in decreased CYP2C6v1 mRNA

**Figure 6:**
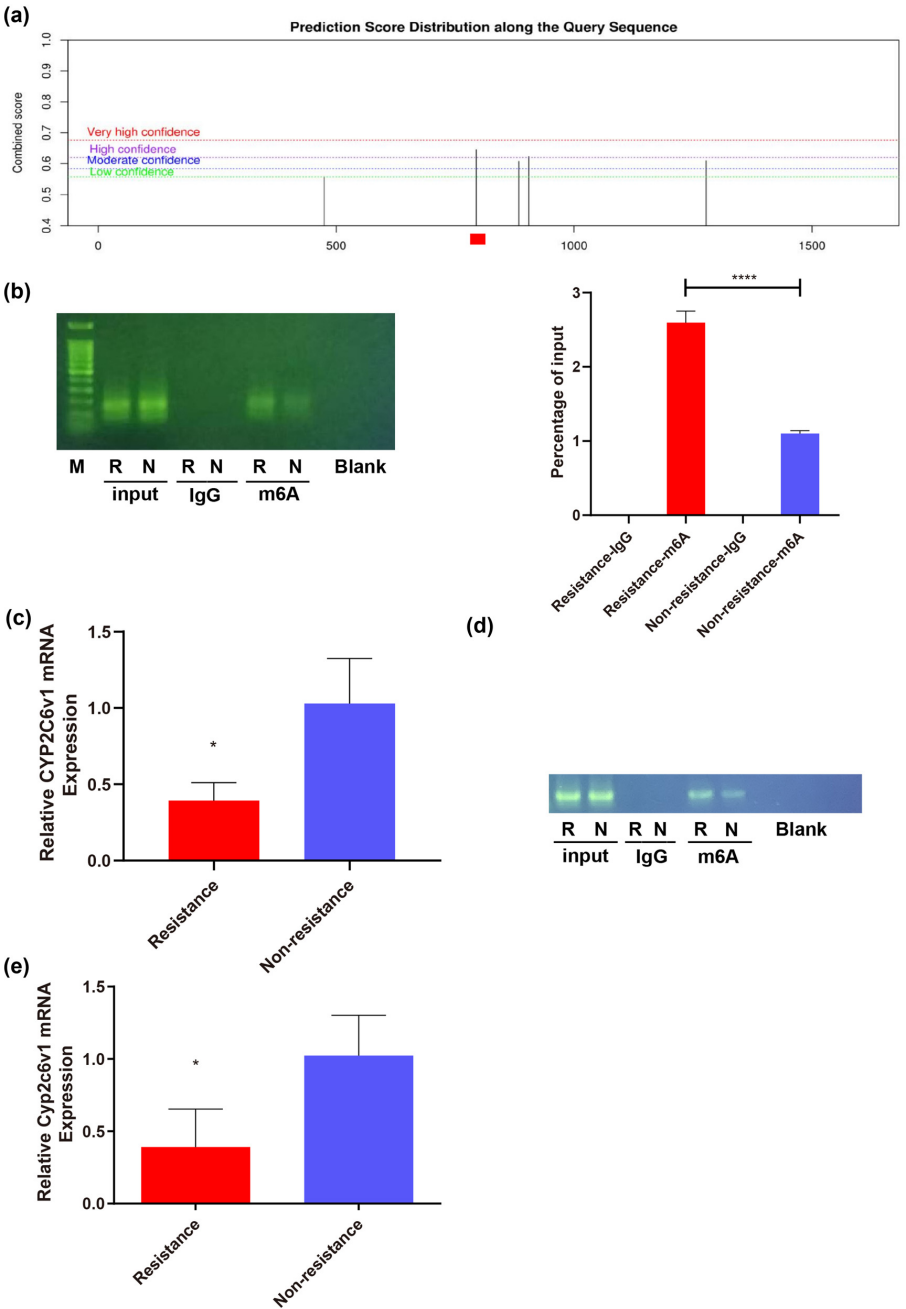
METTL3 modifies CYP2C6v1 RNA in the peripheral blood and liver of rats with CR and results in decreased CYP2C6v1 RNA expression. (a) Prediction of the possible m^6^A methylation site of CYP2C6v1 mRNA in rats. (b) MeRIP-PCR results. m^6^A methylation level of CYP2C6v1 mRNA in the peripheral blood of rats of the clopidogrel-resistant group was significantly higher than the non-clopidogrel-resistant group. (c) qPCR results of CYP2C6v1 expression levels in mRNA in peripheral blood of rats. (d) MeRIP results. M^6^A methylation level of CYP2C6v1 mRNA in liver of rats in clopidogrel-resistant group was higher than that in non-clopidogrel-resistant group. (e) qPCR results of CYP2C6v1 expression levels in mRNA in the liver of rats. Values are mean ± std. *N* = 3. Unpaired *t*-test, *P < 0.05. ****P < 0.0001. R, resistance; N, non-resistance.

Rat CYP2C6v1 is orthologous to human CYP2C19, which activates the metabolism of clopidogrel in rat liver [23]. We made predictions about the possible m^6^A methylation sites for CYP2C6v1 mRNA (Figure 6a). Subsequent MeRIP-PCR results revealed that the m^6^A methylation level of CYP2C6v1 mRNA in the peripheral blood of rats in the CR group was up-regulated 2.6-fold compared to the non-CR group (Figure 6b). Subsequently, the results of qPCR revealed that the expression levels of CYP2C6v1 mRNA in the peripheral blood of rats in the CR group were down-regulated 0.4-fold compared to those in the non-CR group (Figure 6c). Combined, these results along with human MeRIP and qPCR test findings, demonstrate that elevated methylation level of CYP2C19 mRNA/CYP2C6v1 mRNA in peripheral blood correlated with their decreased expression, resulting in CR. Next, to determine whether the CYP2C6v1 mRNA also underwent m^6^A methylation and its expression in the liver, MeRIP and qPCR analysis were performed using RNA samples from rat livers. These results align with those obtained from studies of peripheral blood of rats (Figures 6d and e), suggesting that METTL3 promotes m^6^A methylation at specific sites in CYP2C6v1 mRNA. This methylation leads to reduced expression of CYP2C6v1 mRNA, which in turn results in decreased active clopidogrel metabolism in the liver.

## 4 Discussion

This study found that METTL3 enhanced m^6^A methylation of specific regions of CYP2C19 or CYP2C6v1 mRNA. This increased methylation resulted in reduced expression of CYP2C19 mRNA and protein or CYP2C6v1 mRNA. These changes led to a decrease in the active metabolism of clopidogrel, thereby contributing to the development of CR.

For patients with previous IS, the European Stroke Organization recommends long-term use of antiplatelet therapy to reduce the risk of recurrent stroke [24]. Clopidogrel is a widely used antiplatelet drug, with similar efficacy as aspirin in reducing the risk of IS [25]. However, insufficient inhibition of platelet activation may cause thrombosis progression or recurrence, which may lead to IS progression or relapse [26].

**Figure 7:**
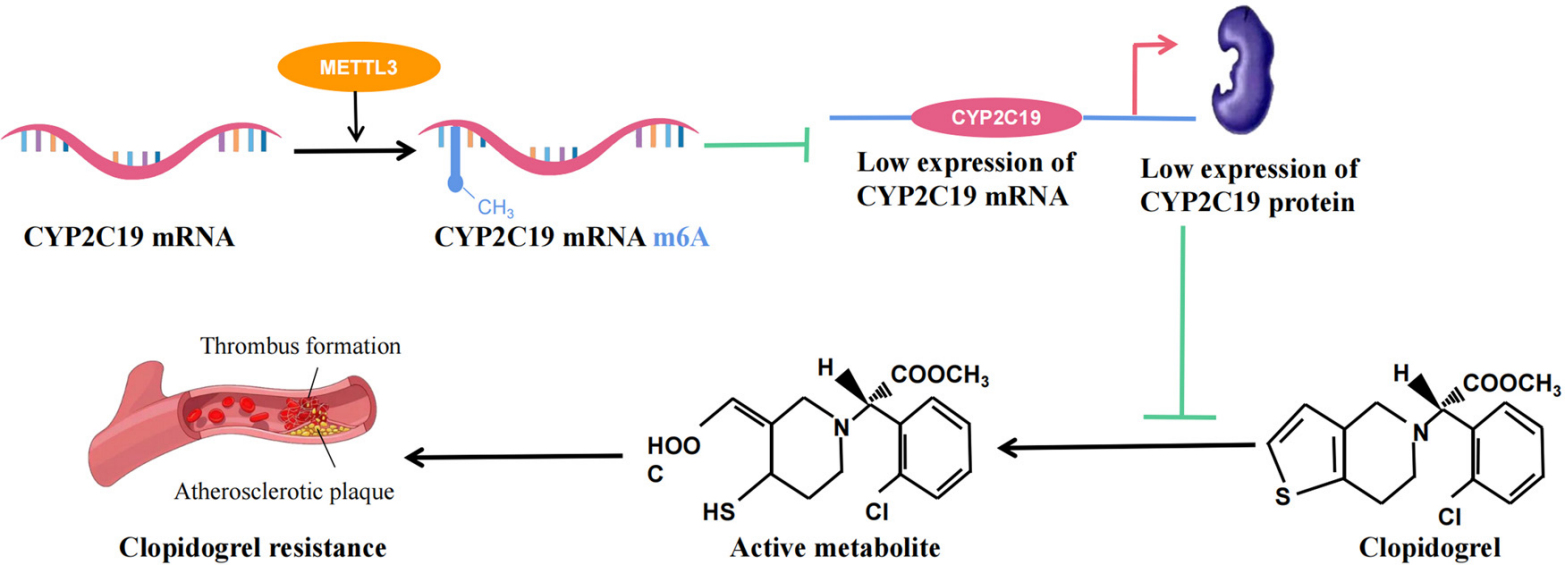
Mechanism by which METTL3 promotes CR in IS by targeting CYP2C19 mRNA N6-methyladenosine.

Previous studies have shown that CR may be affected by genetic polymorphisms, drug interactions, drug dosage, diabetes mellitus, and other factors [8,27–30]. Clopidogrel is a pro-drug that is converted to its active form through the metabolic pathway mediated by the CYP enzyme in the liver, and CYP2C19 is the key enzyme of clopidogrel active metabolism [11]. In addition, previous studies have shown that the polymorphisms of the CYP2C19 gene can affect the efficiency of clopidogrel metabolism, especially the CYP2C19*2 and CYP2C19*3 alleles [31–33]. However, most of the mechanism of CR remains largely unclear. Therefore, studies from other perspectives investigating the possible mechanisms of CR are urgently needed.

m^6^A methylation was first discovered in the 1970s [34], which can influence the function and structure of RNAs, and plays a vital regulatory role in the pathogenesis of multiple diseases, such as cancers, IS, and coronary heart disease [35,36]. In addition, several studies have revealed that m^6^A methylation also plays a part in cancer treatment and drug resistance [15,37]. For example, METTL3 is a main regulator of m^6^A methylation that can lead to resistance to gefitinib, sorafenib, temozolomide, cisplatin, and adriamycin [38–41]. Of note, in our research, we first found that METTL3 was markedly overexpressed in human peripheral blood with CR (Figure 2a). In terms of mechanism, we also showed that CYP2C19 mRNA was affected by m^6^A-dependent methylation of METTL3 (Figure 3) and that this post-transcriptional modification eventually affected the expression of CYP2C19 protein (Figure 3). Therefore, we believe that m^6^A methylation targets CYP2C19 mRNA, and thus contributes to clopidogrel metabolism and CR (Figure 7). Similarly, Nakano et al. showed that METTL3 upregulation could increase m^6^A levels and promote CYP2C8 mRNA degradation, which results in decreased expression of CYP2C8 [18].

CYP2C19 mainly exists in the liver, and the levels of hepatic CYP2C19 and its levels in the peripheral blood are not closely correlated. However, it was difficult for us to obtain CR human liver tissue. The rat CYP2C6v1 gene is homologous to the human CYP2C19 gene, therefore we used rats’ peripheral blood and liver tissue to confirm our hypothesis. First, we completed the construction of the rat model with CR. In this research, CR in rats was defined as having less than a 30% decrease under ADP stimulation in platelet aggregation rate before and after clopidogrel administration [8,42]. Then, in this study, we discovered that the METTL3 levels in the peripheral blood of rats with CR were markedly higher than those of rats with non-CR. In the peripheral blood of rats with CR, METTL3 affected the methylation levels of CYP2C6v1 mRNA, which led to changes in the expression of CYP2C6v1 mRNA. These results corroborated our tests using human peripheral blood with CR. Subsequently, we further verified this result using tissues from the liver of rats. The experimental results in rats’ livers were similar to those in rats’ peripheral blood and human peripheral blood. Meanwhile, we also found that in the liver of rats with CR, the upregulation of METTL3 had led to an increase in the overall levels of m^6^A methylation. Elevated levels of METTL3 lead to an overall increase in global m^6^A levels, resulting in an increase in the methylation levels of CYP2C6v1 mRNA and a decrease in the expression of CYP2C6v1 mRNA. Therefore, it is undeniable that METTL3, the m^6^A writer, is one of the regulators of the expression of the drug-metabolizing enzyme CYP2C6v1 both in the peripheral blood and liver. According to the experimental results, the level of METTL3 and CYP2C19 in peripheral blood can reflect the level of METTL3 and CYP2C19 in the liver, thus they could be useful as new biomarkers for the early and simple diagnosis of CR. Meanwhile, CYP2C19 mRNA m^6^A in the liver might become a new therapeutic target for CR in the future.

However, the findings of this study must take into account the following limitations. First, we used a rat model of CR in this study as a simulation of the common CR phenomenon. The regulation mechanism of CYP2C6v1 mRNA m^6^A in rat liver does not fully reflect the corresponding regulation mechanism of CYP2C19 in human liver. In the future, we will conduct human hepatocyte experiments *in vitro* to further confirm our hypothesis. Second, the exact m^6^A regulatory mechanism of the liver CYP enzyme is still unclear. Shortly, we will conduct animal experiments with overexpression or site-directed knockout to further confirm the exact m^6^A regulating mechanism of CYP enzyme in the liver, and to verify CR’s specific m^6^A targets for diagnosis and intervention.

## 5 Conclusions

In summary, the current study has indicated that METTL3-targeted mRNA methylation regulates the expression of CYP2C19, which in turn leads to individual differences in clopidogrel metabolism and CR. Our findings provide new insights for CR diagnosis and potentially more individualized clopidogrel therapy, warranting further investigations.
